# Comparative Study of TCM Syndrome Scale for Liver Disease and Chronic Liver Disease Questionnaire Based on Assessment of Posthepatitic Cirrhosis

**DOI:** 10.1155/2012/496575

**Published:** 2012-05-29

**Authors:** Hua Zhang, Hua Lv, Pin-Xian Huang, Yan Lin, Xin-Cai Hu, Ping Liu

**Affiliations:** ^1^Key Laboratory of Liver and Kidney Diseases (Ministry of Education), Institute of Liver Diseases, Shuguang Hospital-Shanghai University of Traditional Chinese Medicine, 528 Zhangheng Road, Shanghai 201203, China; ^2^Center for Clinical Effect Evaluation, Shuguang Hospital-Shanghai University of Traditional Chinese Medicine, 528 Zhangheng Road, Shanghai 201203, China; ^3^Department of Preventive Medicine, Shanghai University of Traditional Chinese Medicine, 1200 Cailun Road, Shanghai 201203, China; ^4^Institute of Liver Diseases, Shuguang Hospital-Shanghai University of Traditional Chinese Medicine, 528 Zhangheng Road, Shanghai 201203, China; ^5^E-Institute of Traditional Chinese Internal Medicine, Shanghai Municipal Education Commission, Shanghai University of Traditional Chinese Medicine, 1200 Cailun Road, Shanghai 201203, China

## Abstract

*Objective*. To compare and analyze the relevance and applied value of chronic liver disease questionnaire (CLDQ) and Traditional Chinese Medicine liver disease questionnaire (TCMLDQ) in patients with posthepatitic cirrhosis. *Methods*. The data of 146 patients' scales of CLDQ and TCMLDQ which based on the characteristics of chinese medical symptoms were collected. We made comparative analysis of the relationship between these two scales by the linear regression model and canonical correlation method and evaluated the advantages and disadvantages of two scales about its items setting and dimension definition. *Result*. There is a negative correlation in total scores between the two scales and the linear regression equation: CLDQ = 239.38 − 1.232TCMLDQ. The further canonical correlation analysis was used to analyze the two extracted canonical correlative variables with significances (*P* < 0.05), and the results showed that the overall negative correlation between the two scales mainly came from contributions of both the four dimensions of TCMLDQ (CS, GSYX, GYPX, and OS) and the five dimensions of CLDQ (AS, FA, SS, AC, and EF). *Conclusion*. These two scales have good consistency in the evaluation of severity and life quality of liver cirrhosis patients, so we suggested that TCMLDQ can be used to evaluate the severity and life quality of patients with posthepatitic cirrhosis.

## 1. Background

The questionnaire widely used for assessment of quality of life has been considered as an effective method for quantification, objectification, and standardization of clinical data by World Health Organization, widely recognized by experts, which could be also introduced into the study on quantification of Traditional Chinese Medicine (TCM) symptoms and signs [1]. But how to make the scale design in accordance with TCM theory and its thinking ways accepted by domestic and foreign counterparts and well applied is the key problem to be solved. With selected patients of posthepatitic cirrhosis as research subjects, referring to the basic ideas from assessment quality of life questionnaire, combining with clinical practice and the results based on the study of laws of symptoms and signs classification [2], our task group had preliminarily established TCM liver disease questionnaire (TCMLDQ). Then, through the assessment of both patients and healthy people, TCMLDQ had been confirmed with high reliability, validity, and good sensitivity.

TCM syndromes are the conclusions to the current pathological state of disease made on the basis of synthesis and analysis of information (the body's own feelings and the external appearance) obtained by doctor through the four examinations—inspection, hearing and smelling, inquiring, and palpating. This puts emphasis on the role of individual subjective symptoms in the individualized process of occurrence, development, diagnosis, and treatment of the disease, grasping life and health overall, which has common characteristics with quality of life assessment questionnaire, in order to reflect the advantages and thinking ways of the design of TCM questionnaire and discuss the value and significance of the questionnaire in life quality assessment. In this study, linear regression and canonical correlation analysis methods were used to analyze the comparison of self-developed TCMLDQ and internationally accepted chronic liver disease questionnaire [3] (CLDQ) to explore the relevance between two questionnaires in the evaluation of patient's quality of life and subjective clinical information and provide evidence for recognition and application in counterparts.

## 2. Materials and Methods

### 2.1. Questionnaire

#### 2.1.1. CLDQ (Chinese Version) (See [4])

The questionnaire consists of 6 major categories, 29 questions, and six dimensions as fatigue (FA), activity (AC), emotional function (EF), abdominal symptoms (ASs), systemic symptoms (SSs), and worry (WO) (Table 1). Severities ranged from very serious to no symptoms are divided into 7 classes (1 to 7 points score), and the higher score means the higher quality of life.

#### 2.1.2. TCMLDQ

The questionnaire was self-developed by task group, based on the entry pool constituted preliminary analysis of clinical data of 900 patients with posthepatitis cirrhosis [5]. By pretesting to a little portion of the patients, entries which are repeated, unclearly described, unreadable, or with frequency below 5% were modified or deleted. By reasoning with experts and referring to the CLDQ, TCMLDQ including 38 entries was formed, of which severities ranked from no symptoms to continuous lasting were divided into 7 class (1 to 7 points score), and the higher score indicated the more severe symptoms. By extracting the characteristics of property related to TCM syndromes (similarity analysis to the clinical data of 437 patients with posthepatitis cirrhosis), and combining with the clinical practice and ensuring the uniqueness of the dimension of each entry, five dimensions were classified as common syndromes (CSs, which show commonalities of disease), yin deficiency of liver and kidney (GSYX), yang deficiency of spleen and kidney (PSYX), liver depression, and spleen deficiency (GYPX) and the other syndromes (OSs, symptoms which have no specificities for classification of syndromes) (Table 1).

#### 2.1.3. Evaluating Method for Questionnaire

TCMLDQ and CLDQ were evaluated simultaneously. The investigators are trained in the same way and to unify filling methods and clarify requirement. The two questionnaires are all self-rating scale completed by the patients themselves, and the investigators had given the necessary guidance and instructions to the patients. Score points were marked according to the scoring instruction. 

### 2.2. Clinical Data

All patients were outpatients and inpatients from Shuguang Hospital and Longhua Hospital affiliated to Shanghai University of Traditional Chinese Medicine, Putuo District Center Hospital, and the Shanghai Public Health Clinical Center during the period from 2007 to 2008. 

#### 2.2.1. Recruitment

**Inclusion Criteria**

These include (1) patients who meet the diagnostic criteria of liver cirrhosis (according to “Guide to prevention and treatment of chronic hepatitis B” [6] revised by Liver Diseases Institute, Infectious Diseases institute of Chinese Medical Association in 2005), age 18 to 70 years old, male or female; (2) patient's willingness to participate in scale tests; they can fully understand the significance of scale in all the entries; (3) no previous mental illness history and other psychosomatic disease currently.

**Exclusion Criteria**

These include (1) patients complicated with severe diseases of heart, brain, kidney, lung, endocrine, and hematopoietic system; patients complicated with liver cancer and other serious hepatobiliary diseases and mental illness; (2) patients complicated II degree or above hepatic encephalopathy and severe spontaneous bacterial peritonitis, gastrointestinal bleeding, and hepatorenal syndrome; (3) unclear history of viral infection and other liver diseases related with alcohol, drug, genetic, autoimmune, and so on; (4) women in the period of pregnancy or lactation.

#### 2.2.2. Collection of Clinical Information

A total of 146 patients (average age 46.54 ± 12.54 years) with posthepatitic cirrhosis had been adopted, including 76 inpatients and 70 outpatients; 105 males (average height 171.99 ± 5.25 cm, average weight 67.00 ± 10.82 Kg) and 41 female (average height 159.85 ± 3.96 cm, average weight 59.58 ± 8.85 Kg); 25 cases with a past history of upper gastrointestinal track bleeding; 72 cases with a history of ascites; 77 cases with child-pugh A grade; 45 cases with child-pugh B grade; 24 cases with child-pugh C grade (Table 2).

### 2.3. Statistical Methods

With SPSS17.0 statistical package, the reliability and validity of the TCMLDQ were analyzed by using Cronbach's *α*-coefficient and factor analysis. We carried out an analysis for dependencies between total scores of two scales by using linear regression analysis and introduced the canonical correlation analysis into studying correlation of the two sets of variables (i.e., two scales consisting of different dimensions) and giving a quantitative description of the correlation between two scales.

## 3. Results

### 3.1. The Reliability and Validity of the TCMLDQ

This scale was tested by Cronbach's analysis the **α**-coefficient is 0.844 (more than 0.80), which shows that the internal consistency of entries is good and with high reliability; the assessment of structural validity of the scale was analyzed by factor analysis, the KMO and Bartlett's test showed that *P* value <0.01, so these data were fit for the factor analysis. According to whether the latent root being greater than 1, 14 factors were extracted from 38 entries; the accumulative contribution rate of total variance is 69.45%. The results show that the scale has good structural validity.

### 3.2. Linear Regression Analysis for Total Scores of TCMLDQ and CLDQ Scale

**Figure 1:**
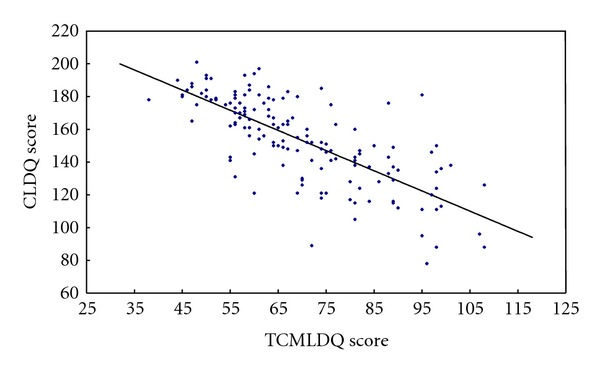
Linear regression plot of total scores of TCMLDQ and CLDQ.

Linear regression analysis was carried out for total scores of 146 patients in two scales to establish regression equation (Table 3, Figure 1). 

From Table 3, the linear regression equation can be drawn: $\text{CLD}\hat{\text{Q}}=239.38-1.232\text{TCMLDQ}$ shows that the total scale score between the two linear correlations was significantly negatively correlated.

**Figure 2:**
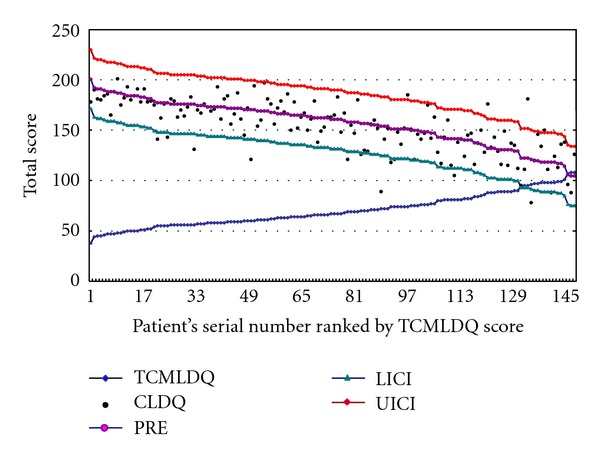
CLDQ total score, predicted values and individual 95% confidence intervals, and TCMLDQ total score line graph. Note: TCMLDQ: TCMLDQ actual measured total score; CLDQ: CLDQ actual measured total score; PRE: CLDQ scores predicted by TCMLDQ score; UICI: upper bounds of predicted CLDQ individual 95% confidence intervals; LICI: lower bounds of predicted CLDQ individual 95% confidence intervals.

We predicted the total score of CLDQ with that of TCMLDQ. Individual 95% confidence intervals is a statistic which reflects the prediction effect of regression equation. It has lower and upper bounds (two predicted total scores of CLDQ) for the prediction interval of the CLDQ for every single case. Through the equation, we can estimate every patient's individual 95% confidence interval of CLDQ and verify whether the actual observation of CLDQ falls in its individual 95% confidence interval. The result has shown that 91.8% of patient's measured values of the CLDQ fall in their corresponding intervals, which means there is a good consistency between TCMLDQ and CLDQ (Figure 2).

### 3.3. Canonical Correlation Analysis between TCMLDQ and CLDQ

We carried out canonical correlation analysis between two sets of dimensions, five dimensions of TCMLDQ as CS (*X*1), GSYX (*X*2), PSYX (*X*3), GYPX (*X*4), and OS (*X*5) and six dimensions of CLDQ as AS (*Y*1), FA (*Y*2), SS (*Y*3), AC (*Y*4), EF (*Y*5), and WO (*Y*6). 

#### 3.3.1. Correlation Analysis between Various Dimensions of TCMLDQ and CLDQ

In addition to having no correlation between *X*3 and *Y*2, *Y*4, *Y*5, *Y*6, TCMLDQ, and CLDQ, the results show negative correlations among the other dimensions (*P* < 0.05) (Table 4).

#### 3.3.2. Extraction of Canonical Correlation Coefficient and Test

This is to discuss whether there is significant correlation in various canonical variables, that is to extract canonical correlation coefficients among canonical variables and carry out hypothesis testing for each pair of canonical correlation coefficients. The results show that there are five pairs of canonical correlation variables; first and second pairs have statistical significant correlation (*P* < 0.05), so these two pairs of canonical correlation variables are selected for analysis (Table 5).

#### 3.3.3. Standardized Correlation Coefficients between Canonical Correlation Variables and Variables of *X* and *Y* Groups

These are Standardized correlation coefficients between *U* canonical correlation variables and various dimensions of TCMLDQ (*X*1 to *X*5), and between *V* canonical correlation variables and various dimensions of CLDQ (*Y*1 to *Y*6) (Table 6). The conversion formula of canonical correlation variable could be written according to 1st to 2nd pairs of canonical variables. 

The formula reflects that the contribution of original variables on canonical variable is determined by the canonical correlation coefficients (i.e., canonical variable loads) between original variables and canonical variables, that is to say, the greater the load capacity, the more impacts on canonical variable by original variable. In accordance with contribution rate to the first pair canonical variable, the original variables follow in the order of *X*4, *X*1, *Y*1, *Y*4, and *Y*5, *Y*2, which means liver depression and spleen deficiency syndrome, common syndrome, and other syndrome in TCMLDQ have the largest contribution to the first pair of extracted canonical correlation variable, while abdominal symptoms, activity, emotional function, and fatigue in CLDQ have the largest contribution to the second pair of extracted canonical correlation variable, and the original variables follow the order of *X*4, *X*1, *X*2, *X*5, and *Y*4, *Y*3, *Y*1, *Y*2, and *Y*5 (correlation coefficient greater than 0.2 [7]), which means liver depression and spleen deficiency syndrome, common syndrome, and yin deficiency syndrome of liver and kidney in TCMLDQ have the larger weight to the second pair of extracted canonical correlation variable, while activity, systematic symptoms, abdominal symptoms, fatigue, and emotional function in CLDQ have larger contribution.

## 4. Discussion

Due to the features of chronic liver diseases—long term, persistent, and recurrent—the therapeutic effects can not simply be evaluated by cure, improvement of laboratory makers, or restoration of normal function, and so forth, in clinic, so comprehensive evaluations of patients' subjective feeling and quality of life were needed. Rating scale or questionnaire is an effective tool for the assessment of respondents' subjective feelings. Subjective symptoms (i.e., the patient's self-feelings) are also the important factors in TCM syndrome differentiation process, which play a main role in identification of TCM syndromes and evaluation of TCM clinical efficacy. But so far, a set of objective methods and standards of evaluating therapeutic effect which can be in line with TCM laws have not been established by TCM. Therefore, Chinese version western scales such as SF-36 [8, 9] and CLDQ [4] were used in evaluation of chronic liver diseases. 

However, the introduction of foreign scale to evaluate the quality of life of Chinese people may cause some misunderstandings due to different cultural background and living habits and could not achieve the goal of syndrome classification in the thinking way of traditional Chinese medicine. For this reason, TCM scholars began referring to psychometric principles and methods to design questionnaires or scale. But no one of scales had gotten the recognition of counterparts in clinical practice. Therefore, we had tried to design TCMLDQ to meet TCM theory and way of thinking and reflect the symptom information and characteristics of syndromes classification of posthepatitic cirrhosis, in order to achieve quantitative assessment of TCM syndromes in posthepatitic cirrhosis. 

TCMLDQ involves a total of five dimensions and 38 entries, common symptoms include 18 entries—fatigue, hypochondriac pain, bitter mouth, halitosis, nausea, yellowish urine, loose stools, difficulty in falling asleep, easy to wake up, dreamfulness, nocturnal enuresis, irritability, depression, skin itching, edema, gum bleeding, epistaxis, and muscle bleeding; other symptoms include headache, dizziness, eye soreness, redden and swollen eyes and throat, dry mouth, belching, dry stool, and night sweating; yin deficiency syndromes of liver and kidney have backache, limb weakness, dry eyes, blurred vision, and tinnitus; liver depression and spleen deficiency syndrome consists of hypochondriac discomfort, abdominal distension, chest and hypochondriac distension, lower abdominal distension, anorexia, and heavy body and limbs; spleen-kidney yang deficiency includes syndrome of aversion to cold and cold limbs. 

At the beginning of this century, CLDQ was introduced to evaluate quality of life and clinical effects for patients with chronic liver disease [10–12], and became a domestic and international accepted specific scale for chronic liver disease, which is used as a reference for the control study with TCMLDQ. CLDQ includes six dimensions and 29 questions. To test different aspects of life quality of patients with chronic liver diseases, its fatigue dimensions consist of sense of fatigue, daytime drowsiness, decreased physical strength, and so forth. Abdominal symptoms include abdominal distension, abdominal pain, abdominal discomfort; activity includes appetite, general weakness, and diet restriction; systemic symptoms include body pain, chest distress, shortness of breath, muscle cramps, dry mouth, and skin itching; emotional function dimension includes anxiety, unhappiness, depression, irritability, sleep disorders, and distraction; worry dimension mainly concentrates on patient's worry with the disease. The different dimensions or categories have a certain degree of overlap, of which the differences in individual experience had been fully taken into account.

CLDQ is used to evaluate the quality of life, and therefore the higher score means the higher quality of life and the milder symptoms. TCMLDQ is used to evaluate the severity of clinical symptoms; the higher score means the more severe symptoms. So considering the results of linear dependencies between total scores of the two scales indicated that there was a significantly negative correlated relationship between the two scales. According to linear relationship between the total score of the two scales, we use the total score of TCMLDQ as independent variables to predict the total score of CLDQ (dependent variable) and make a comparison between predicted and measured scores. The results indicated that the predicted and measured scores had a good match, and almost all observation points were in range of the upper and lower limits of the fitted values. It means that there is a good consistency between TCMLDQ and CLDQ in evaluating the severity of symptoms and quality of life of posthepatitis cirrhosis. 

For further analyzing contribution degree of each dimension to overall correlation of the two scales, we introduced the canonical correlation analysis into study of the linear correlation between two scales. The canonical correlation analysis is used to study the correlation between two sets of multivariables and takes each group of variables as a whole rather than analyzing internal situation in each group of variables. It includes two groups of variables as a whole to find one or more comprehensive variables (linear combination of actual observed variables) to replace original variables, thereby turning the relationship between two sets of variables into the relationship of a few comprehensive variables (canonical variables), which can fully explore the related information between two groups of indicators. 

Canonical correlation analysis was used to analyze the correlation between five dimensions in TCMLDQ and six dimensions in CLDQ. By analyzing the correlation of two groups' dimensions of intersection (interrelations in single dimension), in addition to spleen-kidney yang deficiency and fatigue, activity, emotional function, worry having no correlation, the other showed a negative correlation (*P* < 0.05). Further extracting five pairs of canonical correlation variables, the whole relationship of two groups of dimensions in two scales was analyzed; the overall negative linear correlation mainly comes from negative correlation between the four dimensions of TCMLDQ as common symptoms, yin deficiency syndromes of liver and kidney, liver depression and spleen deficiency syndrome, other symptoms, and five dimensions of CLDQ as abdominal symptoms, fatigue, systemic symptoms, activity, and emotional function (in order of the priority according to the contribution). However, dimension of spleen and kidney yang deficiency syndrome in TCMLDQ and dimension of worry in CLDQ have little or no significant contribution to the overall correlation between the two scales. 

According to the entries and dimensions of two scales, it was believed that there are two aspects of the main factor leading to the results above. First, dimension of spleen-kidney yang deficiency syndrome in TCMLDQ has only one entry of “chills and cold limbs”; there is no such concepts of cold feeling in modern medicine, which are unique evaluation indictors of TCM. So there is no corresponding entry of dimension in CLDQ study, and it is reasonable that this dimension has no contribution to the negative correlation between the two scales. Second, we had a lack of attention on mental, social, and psychological factors in initially prepared TCMLDQ, did not set up the entries to judge the degree of anxiety, and only had two entries associated with irritability and depression. Thus, just like dimension of spleen-kidney yang deficiency syndrome, it is reasonable and realistic that this dimension has no contribution to the negative correlation between the two scales. Therefore, it can also be proved that canonical correlation analysis could be applied into comparison among dimensions of two different scales and could be promoted in the comparison studies of scales in the future.

## 5. Conclusion

According to the results of comparisons between self-developed TCMLDQ and accepted CLDQ scale, TCMLDQ could cover most of the CLDQ's study. They are comparable in dimensions and consistent in the internal structure. That means they could explain and reflect each other to some extent, which had also confirmed that there was a certain rationality for the classification of TCM syndromes based on clinical practice. TCMLDQ described by TCM terms could reflect the quantification of TCM syndromes with TCM characteristics and could also replace CLDQ for the evaluation of severity and life quality of patients with chronic liver disease by continuous improvement and amendments. With improvement of TCM symptoms and signs scale and development and application of instruments and equipment such as tongue diagnosis and pulse-taking diagnosis, it will further improve the quality and level of TCM syndrome evaluation. 

The study focused on analyzing the relationship between the two scales and aimed at laying the methodological foundation for international counterparts.

## Figures and Tables

**Table 1:** The questionnaire dimensionality consists of TCMLDQ and CLDQ.

Dimensionality	Variable	Items	Questions
CLDQ total score	CLDQ	29	AS + FA + SS + AC + EF + WO
Abdominal symptoms (ASs)	*Y*1	3	1, 5, 17
Fatigue (FA)	*Y*2	5	2, 4, 8, 11, 13
Systemic symptoms (SSs)	*Y*3	5	3, 6, 21, 23, 27
Activity (AC)	*Y*4	3	7, 9, 14
Emotional function (EF)	*Y*5	8	10, 12, 15, 16, 19, 20, 24, 26
Worry (WO)	*Y*6	3	18, 22, 25, 28, 29

TCMLDQ total score	TCMLDQ	38	CS + GSYX + GYPX + PSYX + OS
CS	*X*1	18	1, 5, 17, 18, 20, 22, 24, 25, 26, 27, 28, 29, 30, 31, 35, 36, 37, 38
GSYX	*X*2	5	2, 3, 11, 12, 15
PSYX	*X*3	1	33
GYPX	*X*4	6	4, 6, 7, 8, 19, 34
OS	*X*5	8	9, 10, 13, 14, 16, 21, 23, 32

**Table 2:** The general information of patients with posthepatitic cirrhosis.

	Characteristic	Count	Proportion (%)
Patients source	Shuguang Hospital	78	53.42
	Longhua Hospital	56	38.36
	Putuo District Center Hospital	8	5.48
	Shanghai Public Health Clinical Center	4	2.74
Section	Outpatient/inpatient	70/76	47.95/52.05
Sex	Male	105	71.9
	Female	41	28.1
Age (years)	<40	14	9.58
	40–60	105	71.92
	≥60	27	18.49
Virus infection	Hepatitis B virus	143	97.95
	Hepatitis C virus	3	2.05
Splenectomy	Yes	16	10.95

**Table 3:** Linear regression equation of total scores of TCMLDQ and CLDQ.

Model	Unstandardized coefficients		Standardized coefficients	*t*	*P* value	95% confidence interval for *β*	
	*β*	Std. error	Beta			Lower bound	Upper bound
Constant	239.38	6.750		35.462	0.000	226.039	252.724
TCMLDQ	−1.232	0.094	−0.737	−13.069	0.000	−1.418	−1.046

Note: dependent variable: CLDQ total score; TCMLDQ: TCMLDQ total score.

**Table 4:** Correlation coefficients between various dimensions of TCMLDQ and CLDQ.

Dimensions	*Y*1	*Y*2	*Y*3	*Y*4	*Y*5	*Y*6
*X*1	−0.5426**	−0.5711**	−0.5904**	−0.5118**	−0.5578**	−0.3695**
*X*2	−0.2283**	−0.4891**	−0.3352**	−0.2653**	−0.2798**	−0.2001*
*X*3	−0.2171**	−0.0839	−0.2145**	−0.1552	−0.0502	−0.1244
*X*4	−0.6688**	−0.5714**	−0.4349**	−0.6409**	−0.4110**	−0.3430**
*X*5	−0.2416**	−0.3234**	−0.3532**	−0.2115*	−0.3203**	−0.1936*

**Correlation is significant at the 0.01 level (2 tailed).

*Correlation is significant at the 0.05 level (2 tailed).

**Table 5:** Canonical correlation coefficients of variables of TCMLDQ and CLDQ.

Canonical variable	Coefficient	Wilk's	Chi-square	df	*P* value
1 (*U*1 and *V*1)	0.812	0.212	215.318	30	0.000
2 (*U*2 and *V*2)	0.532	0.624	65.619	20	0.000
3 (*U*3 and *V*3)	0.324	0.870	19.418	12	0.079
4 (*U*4 and *V*4)	0.166	0.972	3.988	6	0.678
5 (*U*5 and *V*5)	0.027	0.999	0.103	2	0.950

Note: *U* (*U*1 to *U*5) stands for extracted canonical correlation variables from a group of* X* variables (TCMLDQ); *V* (*V*1 to *V*5) stands for extracted canonical correlation variables from *Y* (CLDQ).

**Table 6:** Standardized *U* and *V* of canonical correlation variables coefficient table.

Variable 1	Standardized correlation coefficients (*U*)					Variable 2	Standardized correlation coefficients (*V*)				
	*U*1	*U*2	*U*3	*U*4	*U*5		*V*1	*V*2	*V*3	*V*4	*V*5
*X*1	0.497	0.749	0.718	0.213	0.814	*Y*1	0.487	−0.674	0.190	0.111	−0.975
*X*2	0.038	0.474	0.972	0.522	0.211	*Y*2	0.222	0.563	−1.233	−0.119	−0.333
*X*3	0.054	0.122	0.453	0.932	0.030	*Y*3	0.094	0.712	0.653	−0.708	0.126
*X*4	0.639	1.056	0.423	0.158	0.341	*Y*4	0.296	−0.792	0.115	0.073	1.055
*X*5	0.135	0.324	0.282	0.314	1.157	*Y*5	0.244	0.438	0.321	1.115	−0.054
						*Y*6	−0.076	0.030	0.122	−0.588	0.372

(1) *U*1 = 0.497*X*1 + 0.038*X*2 + 0.054*X*3 + 0.639*X*4 + 0.135*X*5,

*V*1 = 0.487*Y*1 + 0.222*Y*2 + 0.094*Y*3 + 0.296*Y*4 + 0.244*Y*5 − 0.076*Y*6.

(2) *U*2 = 0.749*X*1 + 0.474*X*2 + 0.122*X*3 + 1.056*X*4 + 0.324*X*5,

*V*2 = −0.674*Y*1 + 0.563*Y*2 + 0.712*Y*3 − 0.792*Y*4 + 0.438*Y*5 + 0.030*Y*6.
